# Factors Associated With Longitudinal Psychological and Physiological Stress in Health Care Workers During the COVID-19 Pandemic: Observational Study Using Apple Watch Data

**DOI:** 10.2196/31295

**Published:** 2021-09-13

**Authors:** Robert P Hirten, Matteo Danieletto, Lewis Tomalin, Katie Hyewon Choi, Micol Zweig, Eddye Golden, Sparshdeep Kaur, Drew Helmus, Anthony Biello, Renata Pyzik, Claudia Calcagno, Robert Freeman, Bruce E Sands, Dennis Charney, Erwin P Bottinger, James W Murrough, Laurie Keefer, Mayte Suarez-Farinas, Girish N Nadkarni, Zahi A Fayad

**Affiliations:** 1 The Dr Henry D Janowitz Division of Gastroenterology Icahn School of Medicine at Mount Sinai New York, NY United States; 2 The Hasso Plattner Institute for Digital Health at Mount Sinai Icahn School of Medicine at Mount Sinai New York, NY United States; 3 Department of Population Health Science and Policy Center for Biostatistics Icahn School of Medicine at Mount Sinai New York, NY United States; 4 Department of Diagnostic, Molecular and Interventional Radiology The BioMedical Engineering and Imaging Institute Icahn School of Medicine at Mount Sinai New York, NY United States; 5 Institute for Healthcare Delivery Science Icahn School of Medicine at Mount Sinai New York, NY United States; 6 Office of the Dean Icahn School of Medicine at Mount Sinai New York, NY United States; 7 Nash Family Department of Neuroscience Icahn School of Medicine at Mount Sinai New York, NY United States; 8 Department of Psychiatry Icahn School of Medicine at Mount Sinai New York, NY United States; 9 The Charles Bronfman Institute for Personalized Medicine Icahn School of Medicine at Mount Sinai New York, NY United States

**Keywords:** wearable device, COVID-19, stress, heart rate variability, psychological, psychology, physiology, mental health, health care worker, observational, app, heart rate, nervous system, resilience, emotion, support, quality of life

## Abstract

**Background:**

The COVID-19 pandemic has resulted in a high degree of psychological distress among health care workers (HCWs). There is a need to characterize which HCWs are at an increased risk of developing psychological effects from the pandemic. Given the differences in the response of individuals to stress, an analysis of both the perceived and physiological consequences of stressors can provide a comprehensive evaluation of its impact.

**Objective:**

This study aimed to determine characteristics associated with longitudinal perceived stress in HCWs and to assess whether changes in heart rate variability (HRV), a marker of autonomic nervous system function, are associated with features protective against longitudinal stress.

**Methods:**

HCWs across 7 hospitals in New York City, NY, were prospectively followed in an ongoing observational digital study using the custom Warrior Watch Study app. Participants wore an Apple Watch for the duration of the study to measure HRV throughout the follow-up period. Surveys measuring perceived stress, resilience, emotional support, quality of life, and optimism were collected at baseline and longitudinally.

**Results:**

A total of 361 participants (mean age 36.8, SD 10.1 years; female: n=246, 69.3%) were enrolled. Multivariate analysis found New York City’s COVID-19 case count to be associated with increased longitudinal stress (*P*=.008). Baseline emotional support, quality of life, and resilience were associated with decreased longitudinal stress (*P*<*.*001). A significant reduction in stress during the 4-week period after COVID-19 diagnosis was observed in the highest tertial of emotional support (*P*=.03) and resilience (*P*=.006). Participants in the highest tertial of baseline emotional support and resilience had a significantly different circadian pattern of longitudinally collected HRV compared to subjects in the low or medium tertial.

**Conclusions:**

High resilience, emotional support, and quality of life place HCWs at reduced risk of longitudinal perceived stress and have a distinct physiological stress profile. Our findings support the use of these characteristics to identify HCWs at risk of the psychological and physiological stress effects of the pandemic.

## Introduction

Increasing rates of SARS-CoV-2 infections and hospitalizations, growing workloads, and concern regarding personal protective equipment have resulted in a large psychological burden on health care workers (HCWs) [1]. Prior pandemics have had psychological effects on HCWs, increasing posttraumatic stress, depression, and anxiety [1-3]. However, the scale and duration of the COVID-19 pandemic have further amplified the risk of these adverse outcomes. Cross-sectional studies have demonstrated that frontline HCWs are at a high risk of depression, anxiety, insomnia, and distress compared to the general population [4-6]. HCWs working in wards serving patients with COVID-19 reported higher levels of stress, exhaustion, depressive mood, and burnout [7,8]. However, there is limited longitudinal data on the pandemic’s psychological impact on this group and across health care occupations, no means to identify which HCWs are at risk of developing psychological sequela over time, and no objective evaluation of the stress response among HCWs. Identification of at-risk HCWs will allow for the appropriate allocation of mental health resources.

Advances in digital technology provide a means to address these limitations. Smartphone apps can be used to administer surveys and integrate wearable devices, such as the Apple Watch, to monitor the autonomic nervous system (ANS), a primary component of the stress response. ANS function can be ascertained through measurement of heart rate variability (HRV), a measure of the parasympathetic and sympathetic nervous systems’ impact on cardiac contractility through calculation of changes in the beat-to-beat intervals [9]. Systems such as the ANS promote adaption to stressors. However, the cumulative burden of stress, or allostatic load, can alter and impair the response of these systems and result in deleterious physical effects on processes ranging from immune function to cardiovascular health. Given the large differences in the response of individuals to stress, an evaluation of both the perceived and physiological consequences of the stressor could provide a more comprehensive overview of its impact and an understanding of who is at risk from the deleterious physical effects of chronic stress [10]. In response to the COVID-19 pandemic, we launched the Warrior Watch Study, comprised of our custom iOS app, which integrates survey metrics with physiological signatures acquired via the Apple Watch. The aim of the study was to understand the longitudinal perceived and physiological stress response among HCWs throughout the course of the pandemic.

## Methods

### Study Design

This was an observational cohort study. The primary objective of the study was to identify characteristics associated with longitudinal stress in HCWs. The secondary aim was to determine whether changes in HRV were associated with features protective against longitudinal stress development. HCWs across 7 hospitals in New York City (The Mount Sinai Hospital, Morningside Hospital, Mount Sinai West, Mount Sinai Beth Israel, Mount Sinai Queens, New York Eye and Ear Infirmary, and Mount Sinai Brooklyn) were eligible. Participants had to be current employees of one of the participating hospitals, ≥18 years of age, have an iPhone Series 6 or higher, and be willing to wear an Apple Watch Series 4 or higher. An underlying autoimmune disease or the use of medications that interfere with ANS function were exclusionary. This study was approved by the institutional review board at Mount Sinai. Informed consent was obtained from all participants.

### Study Procedures

Participants downloaded the custom Warrior Watch Study app to their iPhones and completed eligibility questions prior to signing electronic consent. Through the Warrior Watch Study app, surveys were completed at enrollment and then longitudinally throughout the course of the study. At enrollment, demographic information and whether subjects had a diagnosis of anxiety or depression were collected. Psychological well-being was assessed through structured surveys evaluating perceived stress (Perceived Stress Scale-4 [PSS-4]) [11], resilience (Connor-Davidson Resilience Scale-2 [CD-RISC-2]) [12], emotional support (2-item Patient-Reported Outcomes Measurement Information System [PROMIS] questionnaire) [13], quality of life (2-item Global Health and Quality of Life [QoL]) [14], and optimism (Life Orientation Test) [15] (Table S1, Multimedia Appendix 1). Diagnosis of COVID-19 was defined as a positive SARS-CoV-2 nasal polymerase chain reaction (PCR) swab reported by the study subject.

To longitudinally evaluate subjects, daily surveys were administrated via the study app to collect COVID-19–related symptoms and severity, degree of COVID-19 exposure at work, types of patient care at work, whether participants left their home each day, if public transportation was used, the number of people that participants interacted with each day, the results of any COVID-19 nasal PCR or antibody tests, whether the subject was quarantined, if childcare needs were required, and if the subject was hospitalized. To assess trends in psychological well-being, subjects were prompted to complete the PSS-4 and 2-item General Health and QoL survey weekly. Participants were instructed to wear the Apple Watch for at least 8 hours per day throughout the study period.

### Outcome Measures, Instrumentation, and the Wearable Device

HRV, a physiological marker of stress, was collected via the Apple Watch, while subjective outcome measures were assessed through standardized surveys. The Apple Watch Series 4 or 5 was worn by subjects on the wrist to capture HRV and was connected via Bluetooth to the participants’ iPhone. A photoplethysmogram sensor on the Apple Watch pairs a green LED (light-emitting diode) light with a light-sensitive photodiode to generate time-series peaks [16]. The Apple Watch and Apple Health app calculates HRV using the standard deviation of NN intervals (SDNN) from the time differences between heart beats, categorized as the interbeat interval. SDNN is a time domain index reflecting sympathetic and parasympathetic nervous system activity [9]. This is recorded by the Apple Watch during approximately 60-second recording periods (ultrashort period). Ultrashort term analyses of HRV can be reliably performed to monitor mental health [17]. In accordance with current HRV guidelines, we did not compare SDNN values obtained from recordings of different duration, and we used one measuring device (Apple Watch) to assure standardization of the equipment [18].

The PSS-4 measures perceived stress. It is a 4-question survey that is scored from 0 to 16. Higher scores correlate with elevated perceived stress. The scale evaluates an individual’s confidence in handling problems, whether individuals feel difficulties are piling up, whether things are felt to be going in the way of the individual, and one’s sense of control [11].

The CD-RISC-2 measures resilience. It is a 2-question survey, with higher scores correlating to higher resilience. It includes questions about coping, bouncing back from adversity, whether an individual gets discouraged, and adaptation to change [12].

The 2-item emotional support PROMIS questionnaire is a 2-question survey inquiring about whether individuals have someone to listen to them when they need to talk and someone they trust to discuss their feelings with. It is scored on a scale from 2 to 10 points [13].

The 2-item Global Health and QoL questionnaire asks individuals to grade how their health and quality of life are in general, with higher scores correlating with lower health and quality of life [14].

The Life Orientation Test assesses subject optimism. It comprises 6 questions that ask individuals whether he or she expects the best in uncertain times, whether he or she expects things to go wrong, and whether more good is expected to happen compared to bad [15].

### Statistical Analysis

#### Survey Analyses

To account for gaps created by unanswered weekly surveys and allow comparison for each patient, we created a chronological variable called a “period.” To account for participants having different time windows between each weekly survey, a period was assigned to each weekly survey according to participants’ starting and ending dates. When a participant’s survey was completed less than 7 days from their previous survey date, the day after the previous survey date was regarded as the starting window date for the next period. When a participant’s survey was done 7 days or more apart from the previous survey date, the starting window date was set to 6 days prior to the current survey date. To integrate weekly psychological metrics and daily risk/health metrics, the results of the daily surveys were summarized by the periods defined by the weekly surveys. Daily survey data were summarized for each period (eg, mean number of risk days per period, mean number of days left home per period). To examine the associations between the COVID-19 case count of New York City and perceived stress, raw case count data were obtained for the city and summarized as a mean case count per period [19].

#### Occupation Classification

The occupation of each participant was collected at enrollment. Due to the pandemic, the roles and responsibilities of these occupations may have changed compared to nonpandemic job descriptions. We therefore created a new occupation metric to identify which participants were seeing patients during the study. Occupation was calculated as follows:

1. Daily clinical occupation was calculated from the daily survey where participants classified the type of patient or nonpatient care responsibilities that day. Those who reported either (a) exposure to patient areas but without patients diagnosed with or being evaluated for COVID-19, or (b) exposure to areas with patients confirmed to have COVID-19 or being investigated for COVID-19 infection, were assigned as “clinical” for that day. Those who responded they were at work but not caring for patients or those who were working remotely were classified as “nonclinical” for that day.
2. If a participant had one or more clinical days in a period, that participant was assigned as clinical for that period.
3. If a participant had one or more clinical periods over the entire study, then they were deemed as either clinical nontrainee or clinical trainee. To be classified as a clinical trainee, a participant had to be either a resident or fellow. All other occupations were classified as staff.

#### Statistical Modeling

To model longitudinal changes in stress, we used linear mixed-effect models. Fixed effects included time-invariant covariates (gender, age, occupation, baseline resilience, optimism, and quality of life) and time-variant covariates (COVID-19 diagnosis, SARS-CoV-2/COVID-19 antibody positive test, mobility variables). A continuous first-order autoregressive correlation structure (over period) was found to be suitable for our data, significantly increasing the likelihood function (likelihood ratio test, *P*<*.*001) and leading to a minimal Akaike information criterion or Bayesian information criterion. Model coefficients were estimated using the restricted maximum likelihood approach via R’s *nlme* packages (R Foundation for Statistical Computing). Hypotheses of interest were tested using contrasts through the capabilities of the *emmeans* package.

Univariate models tested the association of each variable with longitudinal stress and identified associated factors. Variables with *P*<*.*10 in the marginal ANOVA (analysis of variance) test were considered significant and included in the multivariate analysis. Although in univariate models random effects include only the intercept, in multivariate models, a random effect for the case burden in New York City was found to be significant (likelihood ratio test, *P*<.001), indicating heterogeneity in the association of this variable with stress across subjects.

#### Heart Rate Variability Modeling

HRV captured from the Apple Watch demonstrated a sparse nonuniform sampling and circadian pattern, making it amenable to analysis via a cosinor model. This approach models the daily HRV circadian rhythm over a period of 24 hours, which can be described using the circadian parameters: (1) midline statistic of rhythm (MESOR), a rhythm-adjusted mean; (2) amplitude, a measure of half the extent of variation within a day; and (3) acrophase, a measure of the time of overall high values recurring in each day. This allows testing of the effect that model covariates have on HRV. A cosinor model used the nonlinear function Y(t)=M + *Acos*(2*π*t/*τ* + *ϕ* + e_i_(t), where *τ* is the period (*τ*=24 hours), M is the MESOR, *A* is the amplitude, and *ϕ* is the acrophase. This can be transformed into the linear model *x*=sin(2*π*t/*τ*), z=sin(2*π*t/*τ*), with HRV written as Y(t)=M + *β*x_t_ + *γ*z_t_ + e_i_(t). We identified a subject-specific daily pattern measuring departures from this pattern as a function of emotional support, resilience, and other covariates of interest. Utilizing a mixed-effect cosinor model HRV, the introduction of random effects intrinsically models the correlation due to the longitudinal sampling. Covariates, C, were introduced as fixed effects using the equation HRV_it_ = M + a_o_Ci + (*β* + a_2_C_i_)x_it_ + (γ + a_3_C_i_)z_it_ + *W_it_θ_i_* + e_i_(t). As we have described previously to test if the cosinor curve differs between two populations of interest, we performed the bootstrapping procedure where for each iteration, we (1) fit a linear mixed-effect model using reweighted least squares, (2) estimated the marginal means for each group defined by a covariate, (3) estimated marginal means for each group using the inverse relationship, and (4) defined the bootstrapping statistics as a pairwise difference between groups [20]. Cosinor models were used to estimate HRV MESOR, amplitude, and acrophase for participants based on emotional support and resilience tertials (low, medium, high). Cosinor model covariates included time, gender, age, BMI, baseline emotional support, baseline resilience, optimism, and stress, with the participant serving as a random intercept.

## Results

### Overview

A total of 361 HCWs were enrolled in this ongoing observational study between April 29 and September 29, 2020, when data were censored for analysis (Table 1). Occupations were classified as (1) clinical nontrainees: participants who reported caring for a patient on any daily survey and was not a resident or fellow, (2) clinical trainee: a resident or fellow, and (3) staff: participants who did not report caring for patients on a daily survey. Participants had a mean age of 37 years, were 69.3% (n=246) female, and were followed for a mean of 60 days (IQR 21-98 days). Clinical trainees had higher baseline resilience compared to clinical nontrainees (*P*=.03) and staff (*P*=.01), higher optimism (*P*=.04) and emotional support (*P*=.01) compared to staff, and higher emotional support compared to clinical nontrainees (*P*=.01) (Table S2, Multimedia Appendix 1).

**Table 1:** Baseline demographic characteristics of the total cohort and by occupation category.

Characteristic			Total cohort (N=361)		Staff (n=65)			Clinical nontrainee (n=217)			Clinical trainee (n=40)	
Age (years), mean (SD)			36.8 (10.1)		36.5 (11.0)			37.8 (10.4)			31.1 (3.6)	
BMI, mean (SD)			25.7 (5.8)		—^a^			—			—	
Female gender, n (%)			246 (69.3)		43 (66.2)			158 (73.8)			20 (51.3)	
**Race, n (%)**												
	Asian	90 (24.9)		14 (21.5)			49 (22.6)			14 (35.0)		
	Black	33 (9.1)		3 (4.6)			23 (10.6)			4 (10.0)		
	White	132 (36.6)		26 (40.0)			80 (36.9)			15 (37.5)		
	Other	47 (13.0)		7 (10.8)			31 (14.3)			6 (15.0)		
**Ethnicity, n (%)**												
	Hispanic	59 (16.3)		15 (23.1)			34 (15.7)			1 (2.5)		
Baseline positive SARS-CoV-2 nasal PCR,^b^ n (%)			22 (6.1)		2 (3.1)			16 (7.4)			2 (5.0)	
Baseline positive SARS-CoV-2 serum antibody, n (%)			35 (9.7)		6 (9.2)			22 (10.1)			2 (5.0)	
**Baseline smoking status, n (%)**												
	Current/past smoker	48 (13.5)		10 (15.4)			31 (14.5)			0 (0.0)		
	Never/rarely smoked	307 (86.5)		55 (84.6)			183 (85.5)			39 (100.0)		
Baseline immune-suppressing medication, n (%)			4 (1.4)		0 (0.0)			4 (1.9)			0 (0.0)	
Anxiety or depression, n (%)			73 (20.6)		16 (24.6)			43 (20.1)			7 (17.9)	
**Baseline survey metrics, mean (SD)**												
	Perceived Stress Scale-4	5.3 (3.1)		5.3 (3.1)			5.5 (2.9)			5.4 (3.1)		
	Connor-Davidson Resilience Scale-2	5.7 (1.4)		5.4 (1.5)			5.7 (1.4)			6.2 (1.3)		
	Optimism	19.1 (4.2)		18.4 (4.3)			18.8 (4.2)			20.1 (3.7)		
	Emotional support	6.8 (1.5)		6.7 (1.7)			6.8 (1.5)			7.6 (0.9)		
	Quality of life	7.8 (1.5)		7.5 (1.4)			7.8 (1.4)			8.0 (1.5)		
**Baseline medical conditions, n (%)**												
	Asthma	41 (11.4)		13 (20)			19 (8.8)			5 (12.5)		
	Chronic lung disease	1 (0.3)		0 (0.0)			0 (0.0)			1 (2.5)		
	Heart disease	1 (0.3)		1 (1.5)			0 (0.0)			0 (0.0)		
	Cancer	2 (0.6)		0 (0.0)			2 (0.9)			0 (0.0)		
	Diabetes mellitus	6 (1.7)		2 (3.1)			4 (1.8)			0 (0.0)		
	Hypertension	20 (5.5)		5 (7.7)			11 (5.1)			0 (0.0)		
	Pneumonia	7 (1.9)		1 (1.5)			5 (2.3)			1 (2.5)		

^a^Not applicable.

^b^PCR: polymerase chain reaction.

### Factors Associated With Longitudinal Stress

**Figure 1:**
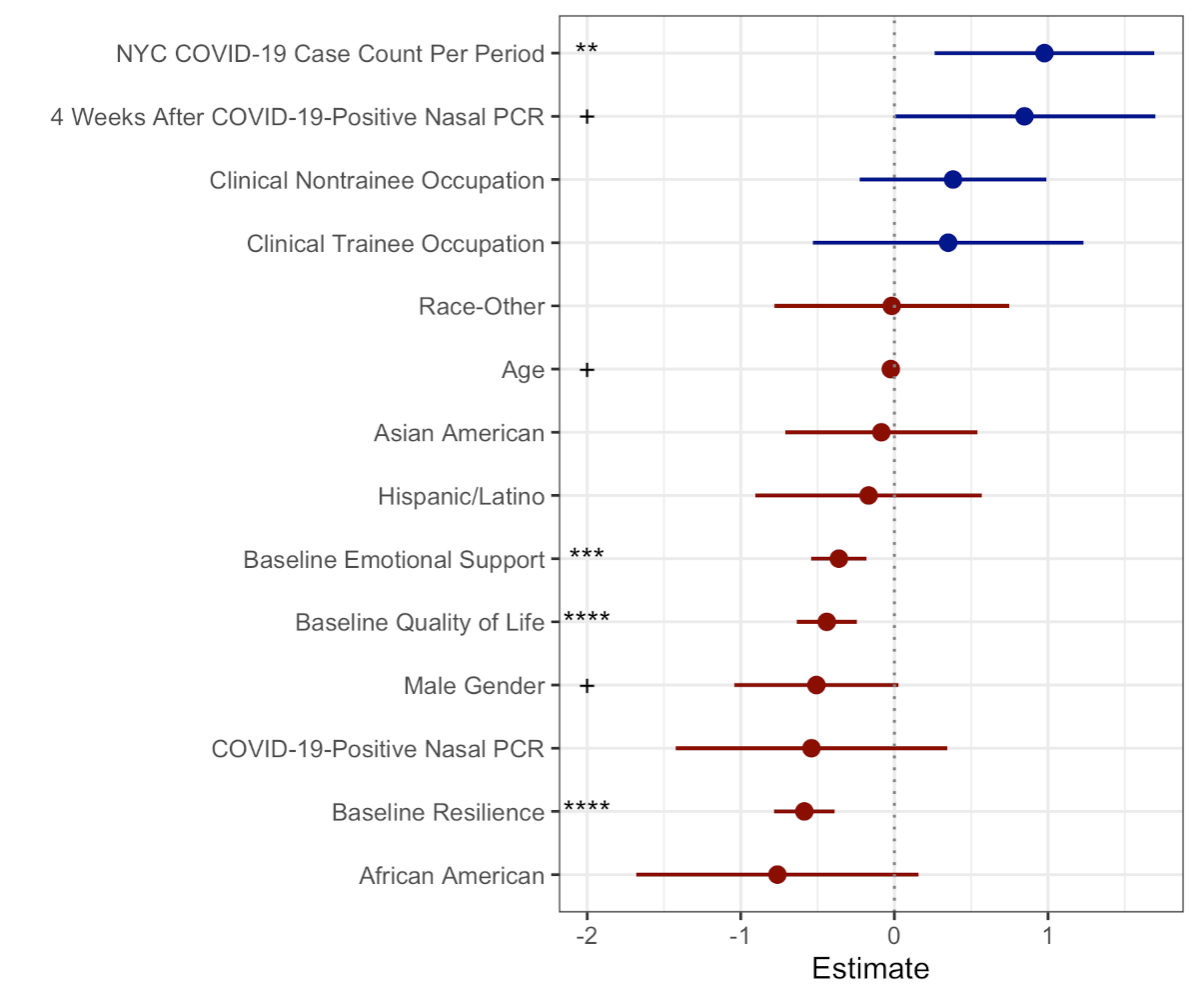
Multivariate analysis of factors associated with longitudinal stress. The scatter plot shows estimated coefficients (CIs) for variables used in the multivariate analysis. Stars indicate that the variable has a significant (*P*<.05) association with longitudinal stress while crosses indicate a borderline significant relationship (*P*<.10). Positive association is indicated in blue and negative association in red. NYC: New York City; PCR: polymerase chain reaction.

The primary aim of the study was to assess the factors associated with longitudinal perceived stress. Univariate analysis evaluated the relationship between baseline demographics and prospectively collected survey metrics with longitudinal perceived stress (Table 2). Baseline factors including resilience, optimism, emotional support, quality of life, male gender, and age were significantly associated with lower longitudinal stress. Baseline anxiety or depression, BMI, weight, and asthma were significantly associated with increased longitudinal stress. Longitudinal quality of life (*P*<.001) was associated with reduced longitudinal stress, while the mean number of COVID-19 cases in New York City (*P*=.004) was positively associated with increased longitudinal stress. Occupation classification (staff vs clinical nontrainee, *P*=*.*81; staff vs clinical trainee, *P*=.15; clinical nontrainee vs clinical trainee, *P*=*.*17), mean number of days caring for patients (*P*=*.*88), and treatment of patients with COVID-19 (*P*=.73) were not associated with longitudinal stress. We observed a significant reduction in stress during the 4-week period following diagnosis (*P*=.01) and over the follow-up period (*P*=.04). Multivariable analysis found only the New York City COVID-19 case count to be significantly associated with increased longitudinal stress (*P*=*.*008). The drop in stress during the 4-week period following COVID-19 diagnosis was not significant (*P*=*.*23); however, we noted a borderline significant increase in stress following the 4-week period after a COVID-19 diagnosis (*P*=*.*05). Baseline emotional support, baseline quality of life, and baseline resilience were associated with decreased longitudinal stress (*P*<*.*001) (Figure 1).

The COVID-19 case count of New York City and the 4-week period after a COVID-19 diagnosis via nasal PCR were further explored in the context of emotional support and resilience. Participants were stratified into emotional support tertials (low, medium, high). A significant reduction in stress during the 4-week period after COVID-19 diagnosis occurred only in participants in the highest tertial of emotional support (effect estimate –0.97, *P*=.03) but not in the medium (effect estimate –0.62, *P*=.48) and low tertials (effect estimate 0.08, *P*=.93) (Figure S1A, Multimedia Appendix 2). A significant trend between New York City’s COVID-19 case count and longitudinal stress was observed only in the high tertial emotional support group (estimate 1.22, *P*=.005), not in the low (estimate –1.45, *P*=.26) or medium (estimate 0.98, *P*=.16) tertials (Figure S1B, Multimedia Appendix 2). Stratification of the cohort into tertials for resilience demonstrated a significant reduction in stress during the 4-week period after COVID-19 diagnosis via nasal PCR in the high (estimate –1.78, *P*=.006) but not medium (estimate 0.33, *P*=.64) and low tertials (estimate –0.60, *P*=.25) (Figure S1C, Multimedia Appendix 2). The impact of New York City’s COVID-19 case count demonstrated a borderline significant relationship with stress in the medium (estimate 1.29, *P*=.098) and high (estimate 1.14, *P*=.09) tertials but not in the low resilience group (estimate 0.72, *P*=.21) (Figure S1D, Multimedia Appendix 2).

**Table 2:** Univariate analysis of factors associated with longitudinal perceived stress.

Factor	Effect estimate	*P* value
Baseline resilience	–0.84	<.001
Baseline optimism	–0.34	<.001
Baseline emotional support	–0.62	<.001
Baseline quality of life	–0.71	<.001
Longitudinal quality of life	–0.80	<.001
Baseline anxiety or depression	1.27	<.001
Baseline BMI	0.07	.001
Male gender	–0.94	.002
Mean New York City case count per period	0.82	.004
No positive COVID-19 nasal PCR^a^ at baseline	0.91	.11
2 weeks post positive COVID-19 nasal PCR test	–0.18	.62
4 weeks post positive COVID-19 nasal PCR test	–0.82	.01
2 weeks post positive COVID-19 antibody test	–0.36	.19
4 weeks post positive COVID-19 antibody test	–0.10	.71
Any period post positive COVID-19 nasal PCR test	–0.85	.04
2 weeks post positive COVID-19 PCR or antibody test	–0.25	.28
Any period post positive COVID-19 antibody test	–0.26	.36
Weight	0.02	.04
Age	–0.03	.047
Baseline asthma	0.89	.045
Baseline heart disease	3.37	.19
Baseline hypertension	–0.34	.58
Baseline diabetes	–0.20	.85
Mean symptomatic days per period	0.32	.07
Staff vs clinical nontrainee	–0.03	.81
Staff vs clinical trainee	0.43	.15
Clinical vs clinical trainee	0.60	.17
Any period post positive COVID-19 PCR or antibody test	–0.47	.07
Height	–0.03	.08
Mean days traveled per period	–0.09	.58
Days left home per period	–0.05	.08
No immune-suppressing medication at baseline	–1.80	.17
No childcare needs at baseline	–0.39	.23
Smoking at baseline	0.53	.20
Mean days hospitalized per period	–1.66	.27
Mean days treating COVID-19–positive patients per period	0.22	.28
Number of days left home	–0.02	.30
Asian vs Black	0.02	.97
Asian vs other	–0.34	.47
Black vs other	–0.36	.55
White vs Asian	0.01	.99
White vs Black	0.03	.96
White vs other	–0.34	.45
No positive COVID-19 antibody test at baseline	0.03	.69
Days caring for patients with COVID-19	0.01	.73
Interacted with 1-3 people outside the home per day	0.01	.97
Interacted with 4-9 people outside the home per day	0.01	.96
Interacted with ≥10 people outside the home per day	–0.11	.64
Mean days quarantined per period	–0.20	.80
Total symptomatic days per period	0.01	.85
Days working weighted based on patient exposure	0.03	.69
Days hospitalized per period	0.07	.88
Days quarantined per period	–0.03	.90
Mean number of days the participant left the house per period	0.03	.90
Mean working days during this period	0.02	.88
Sum of the severity of COVID-19 symptoms per period	–0.001	.96
Mean severity of COVID-19 symptoms this period	0.08	.14

^a^PCR: polymerase chain reaction.

### Physiological Stress Response

**Figure 2:**
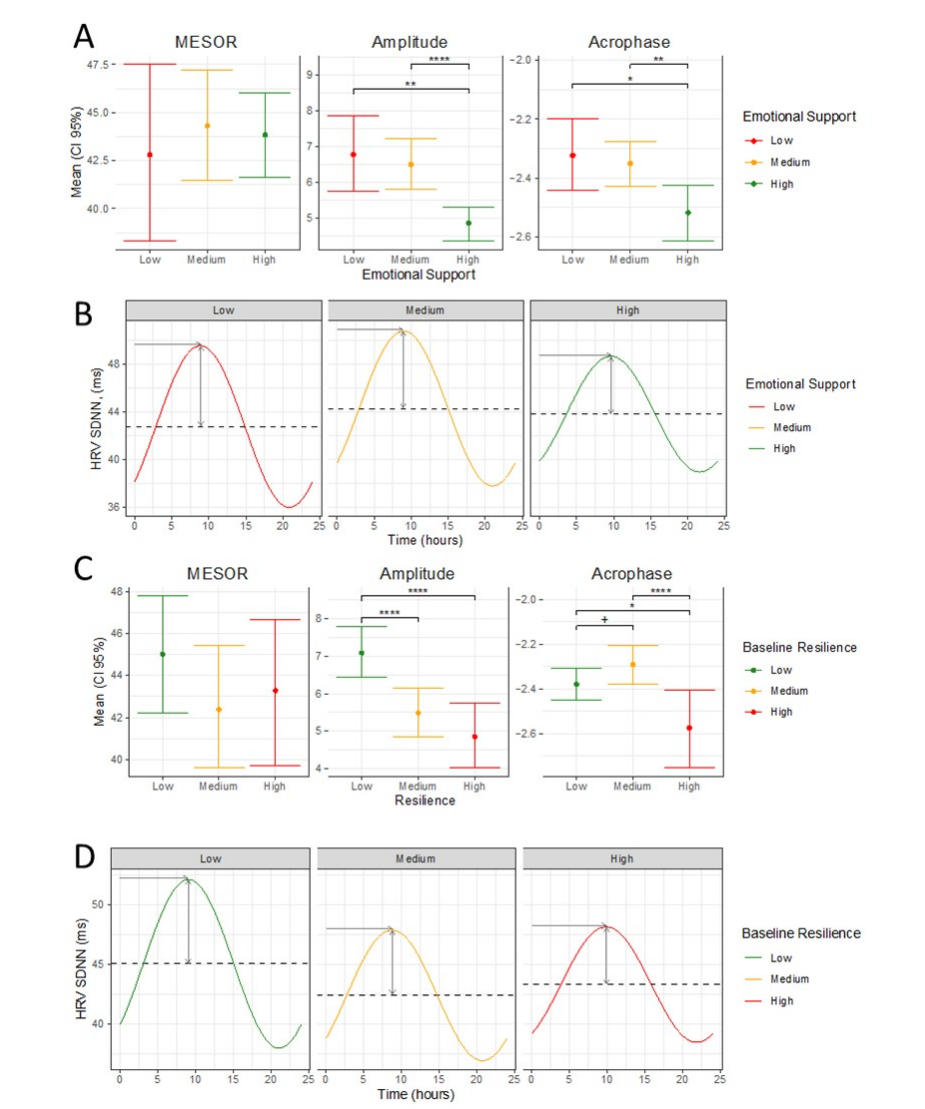
Exploring the relationship between heart rate variability (HRV), emotional support, and resilience. Plots A and C show mean (95% CIs) HRV midline statistic of rhythm (MESOR), amplitude, and acrophase for participants with low, medium, and high emotional support (A) or resilience (C). Stars indicate significant differences between groups. Plots B and D show average daily circadian HRV rhythm for participants with low, medium, and high emotional support (B) or resilience (D). +*P*<.10, *P<.05, **P<.01, ***P<.001. SDNN: standard deviation of NN intervals.

The secondary aim of the study was to evaluate whether features that buffer against perceived stress result in physiological differences in the stress response of HCWs. We fit a cosinor model evaluating differences in HRV (SDNN) (Table S3, Multimedia Appendix 1). A significant reduction in the amplitude of the circadian pattern of SDNN was observed between participants with high compared to medium (*P*<.001) and low (*P*=.008) emotional support (Figure 2A and 2B). There was a significant reduction in the acrophase of the circadian pattern of SDNN in participants with high emotional support compared to those with medium (*P*<*.*001) and low (*P*=.004) emotional support. Significant changes in the circadian pattern of SDNN were also observed when the cohort was stratified based on baseline resilience (Figure 2C and 2D). The amplitude of the circadian pattern of SDNN was significantly lower in subjects with high resilience compared to those with low (*P*<.001) and medium (*P*<.001) resilience. Similarly, the acrophase of participants with high resilience was significantly reduced compared to those with medium (*P*<.001) and low (*P*=.048) resilience (Table 3).

**Table 3:** Comparison of mean heart rate variability parameters stratified based on emotional support and resilience tertials.

Parameter and tertial comparisons			Emotional support, *P* value		Resilience, *P* value
**MESOR^a^**					
	Low vs medium	.60		.10	
	Low vs high	.67		.46	
	Medium vs high	.78		.71	
**Amplitude**					
	Low vs medium	.68		<.001	
	Low vs high	.01		<.001	
	Medium vs high	<.001		.24	
**Acrophase**					
	Low vs medium	.70		.09	
	Low vs high	.004		.048	
	Medium vs high	<.001		<.001	

^a^MESOR: midline statistic of rhythm.

## Discussion

### Principal Results and Comparison With Prior Work

In summary, to the best of our knowledge, we conducted the first study identifying HCW characteristics that correlate with longitudinal stress during the COVID-19 pandemic and identified employees at risk of psychological sequela. We found worsening longitudinal stress to be associated with the number of COVID-19 cases in the community, highlighting the effect of this environmental stressor. Baseline emotional support, resilience, and quality of life, rather than occupation class, defined which HCWs were prone to perceived longitudinal stress and characterized a unique ANS stress profile.

In line with our findings, prior work shows that emotional support and resilience buffer against stress [21,22]. Resilience, defined as a reduced vulnerability to environmental stressors and the ability to overcome difficulty, is crucial to establishing social relationships and is tied to social support, which also acts as an environmental protective factor against adversity [23-25]. In addition to demonstrating their stress protective effect in the multivariate analysis, when we further evaluated the COVID-19 case count of New York City, a factor associated with longitudinal stress over time, we again found that those with lower emotional support or resilience were vulnerable to a dynamic stress response uncoupled from the environmental COVID-19 stressor. Similarly, the transient reduction in stress that occurs after a COVID-19 diagnosis only occurs in those with high emotional support and resilience. Importantly, these latter findings highlight how high resilience or high emotional support can minimize the impact of factors associated with longitudinal stress in HCWs.

A strength of our study is the objective assessment of this observation through longitudinal HRV measurements. HRV is a marker of physiological stress on the ANS. Repeated stressors that are felt to be unmanageable and overwhelming may lead to an impaired stress response characterized by altered autonomic and hypothalamic-pituitary-adrenal axis function [26,27]. Buffers to stress, such as resilience, have also been shown to impact ANS function and are associated with an activation of the sympathetic nervous system that is sufficient to respond to a stressor but not to a degree that results in the development of negative psychological effects [19]. Resilience has been shown to moderate the relationship between perceived stress and the hypothalamic pituitary adrenal axis, which acts as a primary mediator of the physiological stress response [28]. Studies evaluating resilience have demonstrated an associated characteristic ANS profile, with higher vagal indices and with a baseline sympathovagal balance shifted toward parasympathetic predominance [27,29]. Resilience has been associated with the modulation of sympathetic nervous system activation, allowing for an adequate response to a stressor but with moderation of the degree of activation and a rapid return to baseline. This allows for a stable emotional response and reduced chronic anxiety [27,30,31]. Our findings extend these observations into HCWs during the COVID-19 pandemic. We found that buffers of stress, such as high resilience or high emotional support, were characterized by a physiologically distinct ANS profile confirming their impact on how individuals respond to stressors. These findings substantiate the effect these features have on longitudinal stress in HCWs in multiple dimensions, reaffirming their importance not only in the perception of stress but also in how HCWs are physiologically affected by the COVID-19 pandemic stressor.

Our findings have implications for how HCWs can be assessed for the pandemic’s detrimental psychological effects, through screening for an individual’s degree of resilience and social support. Evaluation of these characteristics will assist health care institutions in allocating often limited psychological support services to at-risk individuals. Importantly, one of these features, resilience, is modifiable through targeted interventions. This provides an opportunity to build resilience in HCWs who are found to have low resilience. While further studies are needed on the impact of such interventions in HCWs, they may mitigate the physiological impact of longitudinal stress. Several resilience building interventions have demonstrated to be effective in HCWs [32,33]. However, our findings linking HRV alterations with degree of resilience, makes HRV focused resilience building exercises an attractive option [34].

### Strengths and Limitations

Strengths of the study are its multicenter, longitudinal study design. Furthermore, the number and type of longitudinal variables we captured allowed for a robust multivariate analysis. Lastly, the incorporation of ANS parameters provided an objective assessment of the stress response. However, there are several limitations to our study. The Apple Watch provides HRV data in one-time dimension (SDNN), limiting evaluation of other metrics with outcomes of interests. The Apple Watch also provides HRV sampling sporadically throughout the day. While our modeling accounts for this, a denser sampling would allow expanded analyses. Additionally, HRV can be impacted by many environmental factors that cannot be fully accounted for in such studies. While we tried to control for relevant covariates, there is the potential for unmeasured factors to impact our results.

### Conclusions

We identified features associated with longitudinal perceived stress in HCWs during the COVID-19 pandemic. The COVID-19 case count of New York City was significantly associated with increased longitudinal perceive stress, and baseline emotional support, quality of life, and resilience were associated with decreased longitudinal perceived stress. Furthermore, high resilience and high social support impacted the physiological stress response and were associated with a unique autonomic nervous system profile. This demonstrates the importance of resilience and social support on both perception of stress and its physiological impact.

## Appendix

Multimedia Appendix 1 Supplementary tables.

Multimedia Appendix 2 Supplementary figures. Plots A and C show changes in longitudinal stress following a positive COVID-19 nasal test in participants with low, medium, and high emotional support (A) or resilience (C); stars indicate that change in longitudinal stress was significantly different from zero. Line plots B and D show the relationship between New York City's COVID-19 case count and mean longitudinal stress (CIs) for participants with low, medium, and high emotional support (B) or resilience (D); stars indicate a significant trend between case count and longitudinal stress. +<italic>P</italic><.10, *<italic>P</italic><.05, **<italic>P</italic><.01, ***<italic>P</italic><.001.

## Abbreviations

ANOVA: analysis of variance
ANS: autonomic nervous system
CD-RISC-2: Connor-Davidson Resilience Scale-2
HCW: health care worker
HRV: heart rate variability
LED: light-emitting diode
MESOR: midline statistic of rhythm
PCR: polymerase chain reaction
PROMIS: Patient-Reported Outcomes Measurement Information System
PSS-4: Perceived Stress Scale-4
QoL: quality of life
SDNN: standard deviation of NN intervals
