# Supplementary material for: Factors Associated With Longitudinal Psychological and Physiological Stress in Health Care Workers During the COVID-19 Pandemic: Observational Study Using Apple Watch Data
Source: J Med Internet Res. 2021 Sep 13;23(9):e31295. doi: 10.2196/31295 (PMC8439178; doi:10.2196/31295)
Supplement: Multimedia Appendix 1 [file jmir_v23i9e31295_app1.docx]

**Table S1**. Surveys assessing psychological well-being

| Survey Assessing Wellbeing | Survey Questions | Survey Responses |
| --- | --- | --- |
| Perceived Stress Scale | 1. In the last week, how often have you felt that you were unable to control the important things in your life?  2. In the last week, how often have you felt confident about your ability to handle your personal problems?  3. In the last week, how often have you felt that things were going your way?  4. In the last week, how often have you felt difficulties were piling up so high that you could not overcome them? | Never= 0;  Almost Never= 1; Sometimes= 2;  Fairly Often= 3;  Very Often= 4 |
| Perceived Emotional Support | In the past month, please describe how often:  1. I have someone who will listen to me when I need to talk.  2. I have someone I trust to talk with about my feelings. | Never=1;  Rarely=2;  Sometimes=3;  Usually=4;  Always=5 |
| Resilience Scale | Please indicate how much you agree with the following statements as they apply to you over the last month. If a particular situation has not occurred recently, please answer according to how you think you would have felt.  1. I can deal with whatever comes my way.  2. I am not easily discouraged by failure. | Not true at all= 1;  Rarely True=2;  Sometimes True= 3;  Often True= 4;  True Nearly all the Time= 5 |
| Life Orientation Test | Please be as honest and accurate as you can throughout. Try not to let your response to one statement influence your responses to other statements. There are no "correct" or "incorrect" answers. Answer according to your own feelings, rather than how you think "most people"  would answer.  1. In uncertain times, I usually expect the best.  2. If something can go wrong for me, it will. (R)  3. I'm always optimistic about my future.  4. I hardly ever expect things to go my way. (R)  5. I rarely count on good things happening to me. (R)  6. Overall, I expect more good things to happen to me than bad. | A = I agree a lot;  B = I agree a little;  C = I neither agree nor disagree;  D = I disagree a little;  E = I disagree a lot |
| General Health and Quality of Life | 1. In general, would you say your health is:  2. In general, would you say your quality of life is: | Excellent= 5; Very Good=4; Good= 3; Fair= 2; Poor= 1 |

**Table S2**. Statistical comparison of baseline demographic characteristics.

|  | Effect Estimate | P Value |
| --- | --- | --- |
| Age |  |  |
| Staff vs Clinical Non-Trainee | -1.343 | **0.01** |
| Clinical Non-Trainee vs Clinical Trainee | 6.673 | **<0.001** |
| Staff vs Clinical Trainee | 5.330 | 0.35 |
| Body Mass Index |  |  |
| Staff vs Clinical Non-Trainee | 0.943 | 0.25 |
| Clinical Non-Trainee vs Clinical Trainee | 3.107 | **0.01** |
| Staff vs Clinical Trainee | 4.050 | **0.01** |
| Gender Across Occupations* |  | **0.02** |
| Race Across Occupations* |  | 0.16 |
| Positive SARS-CoV-2 nasal PCR Across Occupations * |  | 0.43 |
| Positive SARS-CoV-2 serum antibody Across Occupations * |  | 0.59 |
| Smoking Status* |  | **0.04** |
| Immune Suppressing Medication* |  | 0.37 |
| Anxiety or Depression* |  | 0.66 |
| PSS-4 |  |  |
| Staff vs Clinical Non-Trainee | 0.041 | 0.93 |
| Clinical Non-Trainee vs Clinical Trainee | 0.418 | 0.44 |
| Staff vs Clinical Trainee | 0.459 | 0.47 |
| CD-RISC |  |  |
| Staff vs Clinical Non-Trainee | -0.255 | 0.20 |
| Clinical Non-Trainee vs Clinical Trainee | -0.510 | **0.03** |
| Staff vs Clinical Trainee | -0.764 | **0.01** |
| Optimism |  |  |
| Staff vs Clinical Non-Trainee | -0.439 | 0.46 |
| Clinical Non-Trainee vs Clinical Trainee | -1.28 | 0.08 |
| Staff vs Clinical Trainee | -1.72 | **0.04** |
| Emotional Support |  |  |
| Staff vs Clinical Non-Trainee | -0.131 | 0.53 |
| Clinical Non-Trainee vs Clinical Trainee | -0.756 | **0.01** |
| Staff vs Clinical Trainee | -0.887 | **0.01** |
| Quality of Life |  |  |
| Staff vs Clinical Non-Trainee | -0.344 | 0.09 |
| Clinical Non-Trainee vs Clinical Trainee | -0.189 | 0.45 |
| Staff vs Clinical Trainee | -0.533 | 0.07 |

*Chi-square test used for statistical comparison

**Table S3**. Mean HRV parameters stratified based upon emotional support and resilience tertials.

| Parameter | Emotional Support Tertial | Mean SDNN Emotional Support Tertial  ms (95% CI) | Resilience Tertial | Mean SDNN Resilience Tertial  ms (95% CI) |
| --- | --- | --- | --- | --- |
| MESOR |  |  |  |  |
|  | Low | 42.80 (37.93-47.42) | Low | 45.03 (41.01-48.08) |
|  | Medium | 44.31 (41.32-47.32) | Medium | 42.38 (39.21-45.49) |
|  | High | 43.8 (41.69-46.05) | High | 43.29 (39.83- 46.64) |
| Amplitude |  |  |  |  |
|  | Low | 6.77 (5.66-7.89) | Low | 7.09 (6.44- 7.80) |
|  | Medium | 6.48 (5.79-7.15) | Medium | 5.49 (4.79- 6.19) |
|  | High | 4.85 (4.35-5.38) | High | 4.85 (3.93-5.67) |
| Acrophase |  |  |  |  |
|  | Low | -2.32 (-2.45- -2.20) | Low | -2.38 (-2.46- -2.31) |
|  | Medium | -2.35 (-2.43- -2.27) | Medium | -2.29 (-2.37- -2.20) |
|  | High | -2.52 (-2.61- -2.43) | High | 2.57 (2.75- -2.40) |
