# Supplementary figures and images for: Factors Associated With Longitudinal Psychological and Physiological Stress in Health Care Workers During the COVID-19 Pandemic: Observational Study Using Apple Watch Data
Source: J Med Internet Res. 2021 Sep 13;23(9):e31295. doi: 10.2196/31295 (PMC8439178; doi:10.2196/31295)

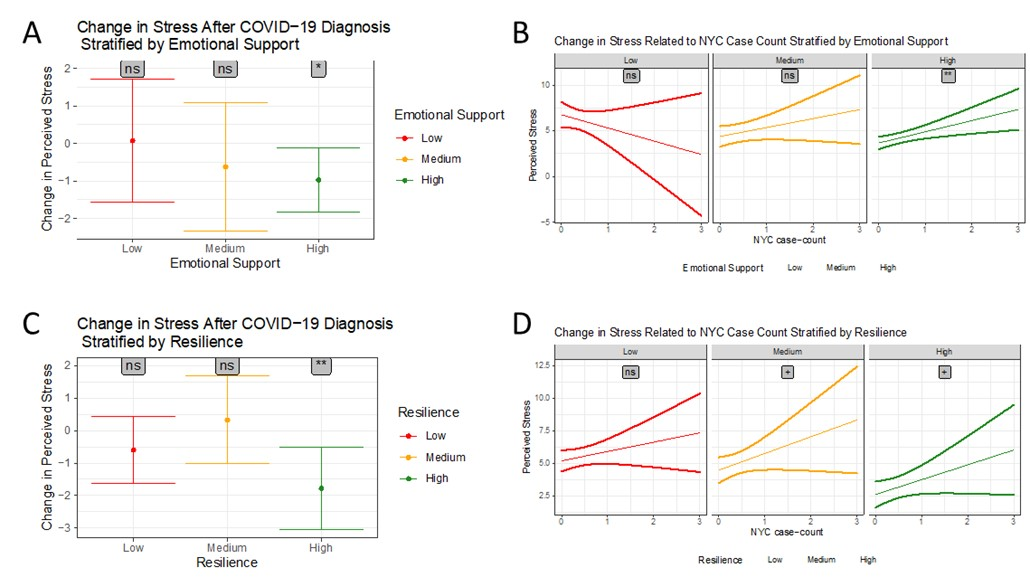

Supplement: Multimedia Appendix 2 [file jmir_v23i9e31295_app2.png]
